# Thyroid function and metabolic syndrome in the population-based LifeLines cohort study

**DOI:** 10.1186/s12902-017-0215-1

**Published:** 2017-10-16

**Authors:** Bruce H. R. Wolffenbuttel, Hanneke J. C. M. Wouters, Sandra N. Slagter, Robert P. van Waateringe, Anneke C. Muller Kobold, Jana V. van Vliet-Ostaptchouk, Thera P. Links, Melanie M. van der Klauw

**Affiliations:** 1 Department of Endocrinology, University of Groningen, University Medical Center Groningen, HPC AA31, P.O. Box 30001, 9700 RB Groningen, The Netherlands; 2 Department of Clinical Chemistry, University of Groningen, University Medical Center Groningen, HPC AA31, P.O. Box 30001, 9700 RB Groningen, The Netherlands

**Keywords:** Metabolic syndrome, Thyroid, Triiodothyronine, Epidemiology

## Abstract

**Background:**

The metabolic syndrome (MetS) is a combination of unfavourable health factors which includes abdominal obesity, dyslipidaemia, elevated blood pressure and impaired fasting glucose. Earlier studies have reported a relationship between thyroid function and some MetS components or suggested that serum free thyroxine (FT4) or free triiodothyronine (FT3) levels within the normal range were independently associated with insulin resistance. We assessed how thyroid function relates to MetS prevalence in a large population-based study.

**Methods:**

Data of 26,719 people of western European descent, aged 18–80 years from the Dutch LifeLines Cohort study, all with normal thyroid stimulating hormone (TSH), FT4 and FT3 levels (electrochemiluminescent immunoassay, Roche Modular E170 Analyzer), were available. MetS was defined with the revised National Cholesterol Education Programs Adults Treatment Panel III (NCEP ATP III) criteria. We calculated prevalence of all MetS components according to TSH, FT4 and FT3 quartiles.

**Results:**

At similar TSH levels and age (mean 45 yrs), men had significantly higher levels of FT4, FT3, blood pressure (BP), heart rate, total and LDL-cholesterol, triglycerides (TG), and creatinine, but lower HDL-cholesterol compared to women (all *p* < 0.001). In total, 11.8% of women and 20.7% of men had MetS. In men, lower FT4 levels were associated with higher prevalence of MetS and all MetS components. In women, lower FT4 quartile was only associated with a higher prevalence of elevated TG, waist circumference, and MetS. However, when corrected for confounding factors like age, BMI, current smoking and alcohol consumption, a significant relationship was found between FT3 and three MetS components in men, and all five components in women. Moreover, the highest quartiles of FT3 and the FT3FT4 ratio predicted a 49% and 67% higher prevalence of MetS in men, and a 62 and 80% higher prevalence in women.

**Conclusions:**

When corrected for possible confounding factors, higher plasma levels of FT3 are associated with several components of the MetS. Only in men, lower FT4 is related to MetS. In the highest FT3 and FT3FT4 quartiles, there is a 50–80% increased risk of having MetS compared to the lowest quartile. Further studies are needed to assess the possible causality of this relationship.

**Electronic supplementary material:**

The online version of this article (10.1186/s12902-017-0215-1) contains supplementary material, which is available to authorized users.

## Background

The metabolic syndrome (MetS) is a clustering of medical conditions that reflects overnutrition, sedentary lifestyles, and resultant adiposity. Metabolic abnormalities such as abdominal obesity, hyperglycaemia, hypertension and dyslipidaemia often are present together, suggesting that they are not independent of one another and that they may share underlying causes and mechanisms [1]. Having MetS places a subject at a substantially increased risk to develop serious diseases like type 2 diabetes (T2D) and cardiovascular disease (CVD) [2].

In an earlier paper we described results from the population-based PREVEND study, and reported that low normal free thyroxine (FT4) levels were significantly related to a higher prevalence of all five MetS components in a group of almost 2300 people from the general population [3]. From this, we concluded that subjects with thyroid function in the low normal range already are at increased cardiovascular risk.

In recent years, there have been confusing results on the relationship between thyroid hormone levels within the normal range and cardiovascular risk factors and MetS. Several papers have described a relationship between thyroid hormone parameters and components of MetS [3–7]. However, some authors have reported an association between thyroid stimulating hormone (TSH) levels and metabolic risk factors, whereas others have observed that high normal free triiodothyronine (FT3) levels, and the FT3FT4-ratio were related to MetS. One of the largest study until now [4] evaluated 44,196 individuals (of whom 25,147 men), and reported that FT4 in men was significantly associated with blood pressure (BP), HDL-cholesterol (HDL-C), fasting blood glucose (FBG), and triglycerides (TG), and in women was associated with BP, HDL-C, FBG, waist circumference (WC). The authors however did not see an association with MetS, when adjusted for age. They did not adjust for smoking or alcohol, two important factors which influence the prevalence of MetS components, like low HDL-C, elevated triglycerides (TG), and -in women- increased WC [8]. Another study from Korea evaluated data from 13,496 middle-aged individuals, and found a relationship between higher T3 and T3/T4 and an unfavourable metabolic profile [6]. And a very recent paper reported in over 132,000 subjects that higher FT3/FT4 ratio was associated with increased risk of metabolic syndrome parameters and insulin resistance, adjusted for age, body mass index, smoking status and menopausal status (in women) [7].

For the current study, we used data of 26,719 participants from the population-based LifeLines Cohort Study to address the discrepancies in the literature. We aimed to investigate the relationship between thyroid hormone parameters and components of the MetS in this large sample of euthyroid subjects in the general population. As differences in MetS prevalence and thyroid hormone parameters have been demonstrated between men and women, we analysed these relationship separately for both sexes.

## Methods

### Subjects

For this cross-sectional study, we obtained data from subjects participating in the LifeLines Cohort Study. LifeLines is a multi-disciplinary prospective population-based cohort study examining in a unique three-generation design the health and health-related behaviours of persons living in the North of The Netherlands. It started in 2007, and employs a broad range of investigative procedures in assessing the biomedical, socio-demographic, behavioural, physical and psychological factors which contribute to the health and disease of the general population, with a special focus on multi-morbidity and complex genetics. The methodology has been described previously [9, 10]. All participants provided written informed consent before entering the study. The study protocol was approved by the medical ethical review committee of the University Medical Center Groningen. For the present study, we included subjects with an age between 18 and 80 years, who were of Western European descent, in whom thyroid hormone parameters were measured, and found to be within the normal range. We excluded participants who were treated because of a thyroid disorder, or who were current users of drugs known to influence thyroid hormone parameters, like lithium, amiodarone, and corticosteroids. Also, participants with a CRP level > 10 mg/L were excluded, because inflammation may disturb the activity of the deiodinase enzymes and alter thyroid hormone levels.

### Clinical examination

Subjects completed a self-administered questionnaire on medical history, past and current diseases, use of medication and health behaviour at home. Medication use was verified by a certified research assistant, and scored by ATC code. The number of different medications used by a participant was considered as a proxy for multimorbidity [11]. Participants were defined as never smoker, current smoker or former smoker. Alcohol intake was calculated as the average number of units of alcohol based on the response to specific questions regarding intake frequency. Individuals who reported that they had not consumed alcohol during the previous month were considered non-drinkers.

BP and anthropometric measurements were obtained using a standardized protocol [9, 10]. BP was measured every minute during a period of 10 min with an automated DINAMAP Monitor (GE Healthcare, Freiburg, Germany). The average of the final three readings was recorded for systolic and diastolic BP. Height, weight, and waist and hip circumference were measured with the participant in light clothing and without shoes [8].

### Biochemical measurements

Blood samples were collected between 8 and 10 a.m. after an overnight fast, directly into tubes containing heparin, and centrifuged. Total cholesterol (TC) and HDL-cholesterol (HDL-C) were measured with an enzymatic colorimetric method, triglycerides (TG) with UV colorometry, and LDL-cholesterol (LDL-C) using an enzymatic method (Roche Modular P chemistry analyser, Roche, Basel, Switzerland). HbA1c was measured with a turbidimetric inhibition immunoassay (Cobas Integra 800 CTS analyser, Roche Diagnostics Nederland BV, Almere, the Netherlands). Fasting blood glucose was measured using a hexokinase method. Levels of thyroid stimulating hormone (TSH), as well as free thyroxine (FT4) and free triiodothyronine (FT3) were assayed by electrochemiluminescent immunoassay (Roche Modular E170, Roche, Switzerland) [12]. Normal values for TSH are 0.4–4.5 mU/l, FT4 are 11–20 pmol/L, for FT3 4.4–6.7 pmol/l. The general Dutch population is iodine sufficient. Tests to measure anti-thyroid peroxidase antibody levels were not performed.

### Calculations, definitions and statistical analysis

Body mass index (BMI) was calculated as weight (kg)/height (m^2^), and categorized as normal weight (<25 kg/m^2^), overweight (25–30 kg/m^2^) and obesity (≥ 30 kg/m^2^). Diagnosis of type 2 diabetes was based either on self-report (known diabetes), or on the finding of a fasting blood glucose ≥7.0 mmol/l at LifeLines’ screening (newly-diagnosed diabetes) [13]. Diagnosis of MetS was established if a subject satisfied at least three out of five criteria according to the modified guidelines of the National Cholesterol Education Programs Adults Treatment Panel III (NCEP ATPIII criteria) [1, 8]. Relevant parameters were calculated according to FT4 and FT3 quartiles. As it is known that women and men differ regarding cardiovascular parameters, as well as MetS and thyroid hormone parameters, all calculations were made for both sexes separately.

Analyses were carried out with PASW Statistics (Version 24, IBM, Armonk, NY, USA). Data are presented as mean ± SD, or median and interquartile range when not normally distributed. Means were compared between groups with analysis of variance. In case of non-normal distribution, medians were compared with the nonparametric Kruskal-Wallis test. Chi-square test was used to analyse categorical variables. Both smoking and alcohol alter various components of the MetS [8]. Multiple linear regression models were therefore created to assess the associations of thyroid hormone parameters with the components of the MetS, with and without correction for factors known to influence either thyroid hormone parameters and MetS components: age, BMI, current smoking, and alcohol consumption. We subsequently used logistic regression analysis to estimate the odds ratios (ORs) and 95% confidence intervals (CI) for quartiles of thyroid hormone parameters to predict the presence of MetS, again adjusted for age, BMI, as well as smoking and alcohol consumption. To allow adjustment for multiple comparisons, a *P*-value <0.001 was considered statistically significant.

## Results

The characteristics of the participants are given for men and women separately in Table 1. At similar TSH levels and age (mean 45 yrs), men had significantly higher levels of FT4, FT3, BP, heart rate, total and LDL-C, TG, and creatinine, but lower HDL-C compared to women (all *p* < 0.001). In total, 11.8% of women and 20.7% of men had MetS.

**Table 1:** Baseline characteristics of the participants

	Men	Women	*P*-value
	*N* = 11.819	*N* = 14.900	
Age (years)	46 ± 13	45 ± 13	<0.001
BMI (kg/m2)	26.5 ± 3.5	25.7 ± 4.4	<0.001
Waist (cm)	96 ± 10	87 ± 12	<0.001
Waist-hip ratio	0.97 ± 0.07	0.87 ± 0.07	<0.001
FT3	5.46 ± 0.53	5.10 ± 0.52	<0.001
FT4	16.1 ± 1.9	15.5 ± 1.8	<0.001
TSH	2.11 ± 0.85	2.20 ± 0.90	<0.001
Normal weight/overweight			
/obesity (n,%)	35.1/51.0/13.9	37.2/47.9/14.9	NS
Current/never smokers (%)	17.5/42.3	33.1/43.4	<0.001/NS
Systolic BP (mmHg)	131 ± 14	122 ± 15	<0.001
Diastolic BP (mmHg)	77 ± 9	72 ± 9	<0.001
Heart rate (b/min)	70 ± 11	72 ± 10	<0.001
Creatinine (mcmol/l)	83 ± 12	68 ± 9	<0.001
Fasting glucose (mmol/l)	5.2 ± 0.8	4.9 ± 0.7	<0.001
HbA1c (%)	5.6 ± 0.4	5.6 ± 0.4	<0.001
Total cholesterol (mmol/l)	5.16 ± 1.00	5.01 ± 0.98	<0.001
HDL-cholesterol (mmol/l)	1.29 ± 0.31	1.60 ± 0.39	<0.001
LDL-cholesterol (mmol/l)	3.38 ± 0.89	3.06 ± 0.88	<0.001
Triglycerides (mmol/l)	1.15 (0.84–1.66)	0.86 (0.65–1.18)	<0.001
% with metabolic syndrome	20.7	11.8	<0.001
% with type 2 diabetes	2.9	1.7	<0.001

In men, lower FT4 levels were associated with lower levels of FT3 and higher TSH levels, higher levels of BMI, WC, waist-to-hip ratio, BP, FBG, total and LDL-C and TG levels, but lower HDL-C. In the lowest FT4 quartile, there were also fewer current smokers. In contrast, lower FT3 quartiles were only associated with lower FT4, higher TSH, and higher age, and also with higher levels of TC, HDL-C and LDL-C, and with a lower percentage of current smokers. As a consequence, lower FT4 levels were associated with higher prevalence of all MetS components. The lowest FT4 quartile (Additional file 1: Table S1) was associated with the highest prevalence of elevated BP (60%), elevated FBG (22.7%), WC (33.4%) and TG (30.2%), low HDL-cholesterol (26.5%), and MetS (27.7%). In contrast, higher FT3 levels were associated only with low HDL-C.

In women, the lowest FT4 quartile was associated with lower FT3 and higher TSH, higher BMI and waist circumference, but not with age, or with differences in blood pressure or cholesterol levels, while triglyceride levels were slightly higher. As a consequence, low FT4 levels (Additional file 2: Table 2) were only associated with elevated TG (11.2%), WC (47.4%), and MetS (13.8%). In contrast, higher FT3 levels were associated only with low HDL-C, and with a higher percentage of participants with ≥3 MetS components.

**Table 2:** Associations of thyroid function with components of the metabolic syndrome in men

		FT4		FT3		TSH		FT3FT4	
	Model	β	P	β	P	β	P	β	P
SBP	1	**−0.048**	**<0.001**	0.008	0.385	0.009	0.330	0.049	**<0.001**
	2	−0.013	0.147	**0.096**	**<0.001**	0.003	0.705	**0.074**	**<0.001**
	3	−0.016	0.084	**0.093**	**<0.001**	0.003	0.741	**0.075**	**<0.001**
	4	0.015	0.098	**0.081**	**<0.001**	0.005	0.576	**0.040**	**<0.001**
DBP	1	**−0.048**	**<0.001**	**−0.047**	**<0.001**	−0.017	0.066	0.009	0.304
	2	−0.005	0.584	**0.054**	**<0.001**	−0.024	0.007	**0.039**	**<0.001**
	3	−0.006	0.486	**0.052**	**<0.001**	−0.027	0.002	**0.039**	**<0.001**
	4	0.020	0.023	**0.041**	**<0.001**	−0.026	0.003	0.009	0.312
HDL-C	1	**0.044**	**<0.001**	**−0.097**	**<0.001**	−0.016	0.086	**−0.112**	**<0.001**
	2	**0.060**	**<0.001**	**−0.072**	**<0.001**	−0.018	0.048	**−0.103**	**<0.001**
	3	**0.065**	**<0.001**	**−0.063**	**<0.001**	**−0.032**	**<0.001**	**−0.100**	**<0.001**
	4	0.023	0.008	**−0.045**	**<0.001**	**−0.035**	**<0.001**	**−0.051**	**<0.001**
TG	1	**−0.084**	**<0.001**	0.011	0.246	**0.057**	**<0.001**	**0.083**	**<0.001**
	2	**−0.074**	**<0.001**	0.040	**<0.001**	**0.076**	**<0.001**	**0.092**	**<0.001**
	3	**−0.081**	**<0.001**	0.027	0.005	**0.068**	**<0.001**	**0.090**	**<0.001**
	4	−**0.040**	**<0.001**	0.010	0.286	**0.070**	**<0.001**	**0.041**	**<0.001**
FBG	1	**−0.074**	**<0.001**	**−0.040**	**<0.001**	−0.003	0.710	**0.038**	**<0.001**
	2	**−0.042**	**<0.001**	**0.037**	**<0.001**	−0.009	0.333	**0.062**	**<0.001**
	3	**−0.043**	**<0.001**	**0.036**	**<0.001**	−0.008	0.353	**0.062**	**<0.001**
	4	−0.014	0.112	0.024	0.010	−0.007	0.456	0.028	**0.001**
Waist	1	**−0.143**	**<0.001**	−0.018	0.051	−0.015	0.106	**0.119**	**<0.001**
	2	**−0.105**	**<0.001**	**0.079**	**<0.001**	−0.021	0.016	**0.148**	**<0.001**
	3	**−0.106**	**<0.001**	**0.079**	**<0.001**	−0.021	0.017	**0.148**	**<0.001**
	4	−0.002	0.657	**0.036**	**<0.001**	−0.015	0.001	**0.0125**	**<0.001**

Values of β are standardized regression coefficients

Regression for triglycerides was performed after logarithmic transformation. Significant associations are shown in bold

Model 1: crude

Model 2: after adjustment for age

Model 3: after adjustment for age, current smoking, alcohol consumption

Model 4: after adjustment for age, BMI, current smoking, alcohol consumption

It became apparent that age, BMI and current smoking were confounders in all analyses: higher age was associated with lower FT3, and the lower FT4 and FT3 quartiles contained fewer current smokers. Also, age, BMI, smoking and alcohol consumption are known to influence several MetS components. Therefore, we performed four models within linear regression analyses, in which model 4 corrects for all these confounders. The results of these analyses are depicted in Tables 2 and 3, and show some differences between men and women. In men, only the association between FT4 and TG remained significant after correcting for all confounders, while FT3 correlated with 3 of 5 MetS components (BP, HDL-C, WC). In women, FT3 was associated with all MetS components after correction for the confounders. In both men and women, the FT3FT4-ratio was associated 4 of the 5 components of MetS, but not with FBG.

**Table 3:** Associations of thyroid function with components of the metabolic syndrome in women

		FT4		FT3		TSH		FT3FT4	
	Model	β	P	β	P	β	P	β	P
SBP	1	**0.034**	**<0.001**	**0.040**	**<0.001**	**0.033**	**<0.001**	0.006	0.455
	2	**0.027**	**<0.001**	**0.131**	**<0.001**	0.025	0.001	**0.081**	**<0.001**
	3	**0.028**	**<0.001**	**0.137**	**<0.001**	0.023	0.002	**0.083**	**<0.001**
	4	**0.054**	**<0.001**	**0.130**	**<0.001**	0.018	0.015	**0.054**	**<0.001**
DBP	1	−0.003	0.754	0.015	0.06	0.007	0.426	0.017	0.044
	2	−0.007	0.400	**0.064**	**<0.001**	0.002	0.823	**0.058**	**<0.001**
	3	−0.006	0.461	**0.068**	**<0.001**	0.000	0.962	**0.059**	**<0.001**
	4	0.014	0.078	**0.063**	**<0.001**	−0.004	0.658	**0.037**	**<0.001**
HDL-C	1	0.016	0.046	**−0.112**	**<0.001**	0.020	0.016	**−0.106**	**<0.001**
	2	0.013	0.107	**−0.079**	**<0.001**	0.016	0.047	−0.078	<0.001
	3	0.023	0.005	**−0.062**	**<0.001**	0.001	0.877	**−0.071**	**<0.001**
	4	−0.016	0.034	**−0.052**	**<0.001**	0.009	0.240	−0.027	<0.001
TG	1	**−0.068**	**<0.001**	**0.046**	**<0.001**	**0.078**	**<0.001**	**0.105**	**<0.001**
	**2**	**−0.073**	**<0.001**	**0.101**	**<0.001**	**0.073**	**<0.001**	**0.154**	**<0.001**
	**3**	**−0.083**	**<0.001**	**0.086**	**<0.001**	**0.087**	**<0.001**	**0.150**	**<0.001**
	4	**−0.051**	**<0.001**	**0.078**	**<0.001**	**0.080**	**<0.001**	**0.113**	**<0.001**
FBG	1	0.000	0.964	−0.011	0.161	**0.036**	**<0.001**	−0.003	0.731
	2	−0.007	0.405	**0.059**	**<0.001**	**0.029**	**<0.001**	**0.057**	**<0.001**
	**3**	−0.007	0.405	**0.059**	**<0.001**	**0.029**	**<0.001**	**0.056**	**<0.001**
	4	0.025	0.001	**0.051**	**<0.001**	0.023	0.002	0.020	0.011
Waist	1	**−0.091**	**<0.001**	−0.011	0.175	0.018	0.032	**0.081**	**<0.001**
	2	**−0.096**	**<0.001**	**0.048**	**<0.001**	0.012	0.137	**0.134**	**<0.001**
	3	**−0.097**	**<0.001**	**0.047**	**<0.001**	0.014	0.084	**0.133**	**<0.001**
	4	−0.004	0.380	**0.024**	**<0.001**	−0.005	0.284	**0.024**	**<0.001**

Values of β are standardized regression coefficients

Regression for triglycerides was performed after logarithmic transformation. Significant associations are shown in bold

Model 1: crude

Model 2: after adjustment for age

Model 3: after adjustment for age, current smoking, alcohol consumption

Model 4: after adjustment for age, BMI, current smoking, alcohol consumption

Finally, Tables 4 and 5 depicts the results of the logistic regression analyses, in which we have correlated the thyroid parameters (divided in quartiles again) with the presence of MetS. In both men and women, higher levels of FT3 and the FT3FT4-ratio, adjusted for age, BMI, current smoking and alcohol consumption, were associated with a higher risk MetS, while FT4 was associated with higher risk of MetS in men only. Both for men and women, we observed a 1.67–1.80 higher OR for the highest FT3FT4-ratio quartile compared to the lowest quartile to predict the presence of MetS.

**Table 4:** Risk of having metabolic syndrome according to quartiles of thyroid hormone parameters, in men

	Quartile	MetS/Total, n (%)	model 1	model 2	model 3	model 4
TSH	1	578/2923 (19.8)	1	1	1	1
	2	595/2975 (20.0)	1.014 (0.893–1.153)	1.015 (0.892–1.155)	1.030 (0.904–1.174)	1.000 (0.861–1.161)
	3	618 (2946 (21.0)	1.077 (0.948–1.223)	1.072 (0.943–1.219)	1.116 (0.980–1.271)	1.101 (0.949–1.276)
	4	650/2975 (21.8)	1.134 (1.000–1.286)	1.110 (0.977–1.261)	1.155 (1.015–1.314)	1.214 (1.047–1.407)
FT4	1	796/2877 (27.7)	1	1	1	1
	2	635/3000 (21.2)	**0.702 (0.623–0.791) #**	**0.717 (0.635–0.809) #**	**0.704 (0.623–0.796) #**	**0.816 (0.709–0.939) #**
	3	532/3014 (17.7)	**0.560 (0.495–0.635) #**	**0.596 (0.525–0.675) #**	**0.579 (0.509–0.657) #**	**0.733 (0.634–0.848) #**
	4	478/2928 (16.3)	**0.510 (0.449–0.579) #**	**0.567 (0.498–0.646) #**	**0.545 (0.478–0.622) #**	**0.722 (0.621–0.839) #**
FT3	1	516/2655 (19.4)	1	1	1	1
	2	652/3300 (19.8)	1.021 (0.897–1.161)	1.192 (1.045–1.360)	**1.172 (1.026–1.339) #**	1.174 (1.010–1.366)
	3	695/3185 (21.8)	1.157 (1.018–1.315)	**1.487 (1.302–1.699) #**	**1.458 (1.275–1.668) #**	**1.360 (1.167–1.586) #**
	4	578/2679 (21.6)	1.140 (0.998–1.303)	**1.652 (1.434–1.904) #**	**1.577 (1.366–1.821) #**	**1.491 (1.266–1.756) #**
FT3FT4	1	457/2945 (15.5)	1	1	1	1
	2	528/2958 (17.8)	1.183 (1.031–1.357)	1.241 (1.080–1.426)	**1.220 (1.060–1.405) #**	1.234 (1.054–1.445)
	3	667/2960 (22.5)	**1.584 (1.388–1.807) #**	**1.753 (1.533–2.005) #**	**1.731 (1.512–1.983) #**	**1.537 (1.318–1.791) #**
	4	789/2956 (26.7)	**1.982 (1.743–2.255) #**	**2.263 (1.984–2.582) #**	**2.267 (1.984–2.590) #**	**1.674 (1.438–1.948) #**

Model 1: crude; model 2: adjusted for age; model 3: adjusted for age, current smoking and alcohol consumption

model 4: adjusted for age, BMI, current smoking and alcohol consumption

OR and 95% CI for metabolic syndrome were calculated using logistic regression models

Significant associations (P < 0.001) are shown in bold

**Table 5:** Risk of having metabolic syndrome according to quartiles of thyroid hormone parameters, in women

	Quartile	MetS/Total, n (%)	model 1	model 2	model 3	model 4
TSH	1	388/3726 (10.4)	1	1	1	1
	2	428/3710 (11.5)	1.122 (0.970–1.298)	1.149 (0.989–1.334)	1.192 (1.025–1.386)	1.162 (0.984–1.372)
	3	451/3729 (12.1)	1.184 (1.025–1.367)	1.165 (1.005–1.351)	1.219 (1.049–1.417)	1.046 (0.885–1.236)
	4	493/3735 (13.2)	1.308 (1.136–1.507) #	1.212 (1.053–1.061)	1.288 (1.110–1.494)	1.167 (0.990–1.375)
FT4	1	523/3784 (13.8)	1	1	1	1
	2	417/3707 (11.2)	0.790 (0.689–0.907)	**0.761 (0.660–0.876) #**	**0.757 (0.656–0.874) #**	0.854 (0.728–1.002)
	3	423/3624 (11.7)	0.824 (0.718–0.945)	**0.762 (0.661–0.877) #**	**0.758 (0.657–0.874) #**	0.900 (0.767–1.057)
	4	397/3785 (10.5)	**0.731 (0.636–0.840) #**	**0.651 (0.564–0.752) #**	**0.620 (0.535–0.717) #**	0.819 (0.696–0.964)
FT3	1	317/3104 (10.2)	1	1	1	1
	2	482/4361 (11.1)	1.095 (0.943–1.272)	1.179 (1.011–1.375)	1.154 (0.988–1.348)	1.095 (0.923–1.299)
	3	521/3897 (13.4)	**1.357 (1.170–1.574) #**	**1.644 (1.410–1.916) #**	**1.558 (1.334–1.819) #**	**1.515 (1.276–1.798) #**
	4	439/3538 (12.4)	1.245 (1.068–1.452)	**1.819 (1.550–2.134) #**	**1.707 (1.451–2.008) #**	**1.622 (1.354–1.942) #**
FT3FT4	1	374/3724 (10.0)	1	1	1	1
	2	392/3718 (10.5)	1.056 (0.909–1.226)	1.227 (1.051–1.432)	1.217 (1.041–1.423)	1.241 (1.045–1.473)
	3	438/3726 (11.8)	1.193 (1.031–1.381)	**1.557 (1.337–1.813) #**	**1.539 (1.319–1.795) #**	**1.423 (1.201–1.687) #**
	4	556/3732 (14.9)	**1.568 (1.364–1.803) #**	**2.372 (2.045–2.751) #**	**2.329 (2.004–2.705) #**	**1.798 (1.522–2.125) #**

Model 1: crude; model 2: adjusted for age; model 3: adjusted for age, current smoking and alcohol consumption

model 4: adjusted for age, BMI, current smoking and alcohol consumption

OR and 95% CI for metabolic syndrome were calculated using logistic regression models

Significant associations (P < 0.001) are shown in bold

## Discussion

In this large population-based study in subjects with thyroid hormone levels in the normal range, we have shown that higher levels of FT3 and the FT3FT4 ratio and lower levels of FT4 (in men only) are associated with components of MetS, when corrected for important confounders like age, BMI, current smoking and alcohol consumption. A significant relationship was found for FT3 and three MetS components in men, and all five components in women. Moreover, the highest quartiles of FT3 and the FT3FT4 ratio predicted a 49% and 67% higher prevalence of MetS in men, and a 62 and 80% higher prevalence in women.

From our uncorrected analyses, FT4 levels appeared the most strongly related to MetS. However, it has been known that both age and BMI can influence thyroid hormone parameters and MetS. In the current study, we observed a gradual decrease of plasma FT3 levels with increasing age, while TSH and FT4 levels did not change significantly. Furthermore, it is known that FT4 levels are lower and TSH levels are higher in obese individuals compared to those with normal weight. This was the reason we included both parameters as confounders in our linear regression models. In addition, both smoking and alcohol consumption have a profound effect on some MetS components. Smoking lowers HDL-C levels, and is associated with increased WC, as we have demonstrated earlier within the LifeLines population [8]. Alcohol consumption may increase HDL-C levels, but also levels of BP [8, 14, 15]. As our initial data analyses revealed that there were fewer smokers and users of alcohol in the lowest FT4 and FT3 quartiles, both in men and in women, we have corrected in our regression models for these factors as well. And only with these important adjustments, we were able to demonstrate that FT3 levels and the FT3FT4 ratio were significantly associated with MetS components, and having MetS.

Several papers have described the relationship between thyroid hormone parameters and components of MetS. In 2007, our group has demonstrated in the population-based PREVEND study the association between levels of FT4 (within the normal reference range) and plasma lipids; these findings were in accordance with the earlier observed association between (sub)clinical hypothyroidism and hyperlipidaemia [3]. Low normal FT4 levels proved to be significantly associated with MetS (four of five components) and increased insulin resistance. From our data, we concluded that this suggested an increased risk of cardiovascular events in subjects with low normal thyroid function [3]. Several additional papers, as summarized in Table 6, have been published the last decade which have assessed the relationship between thyroid hormone levels and cardiovascular risk factors, including the MetS. These studies have been performed in populations with different ethnic background. As can be seen in Table 6, there are significant differences between these studies, not only related to study setting and population, but also related to the results. One of the striking differences is that several papers have not separated between women and men, as it is known (which is also apparent from the current paper) that the prevalence of the individual MetS components differs considerably between women and men [16–19]. For instance, we have recently demonstrated that increased waist circumference is a common features of MetS among women while men have often an elevated blood pressure [20]. Moreover, FT4 and FT3 levels differ between men and women as well. Therefore, simply adjusting for sex may obscure possible differences between sexes. In addition, no study has reported data on smoking and alcohol consumption, or corrected for these important confounders. Interestingly, our data indicate a dysbalance of smoking and alcohol consumption in the different FT4 and FT3 quartiles, which causes a warning regarding the proper interpretation of earlier studies. With the exception of recent studies by Kim et al. [4], Park et al. [5] and by Kim et al. [6, 21], earlier studies have incorporated a limited number of participants.

**Table 6:** Studies evaluating the relationship between thyroid hormone levels and components of the metabolic syndrome

Author, year	N	Subjects’ age	Gender separation?	Corrections for confounders	Major findings	Ref
Roos, 2007	1581, 716 M	28–75 yrs	No	Age, sex, HOMA-IR	FT4 negative association with TC, LDL-C, TG and waist, positive association with HDL-C, FT3 negative association with TC, LDL-C, TG	[3]
Park, 2009	949	Postmeno-pausal women	Women only	Age, BMI, HOMA-IR, FT4, exercise (3 levels), alcohol (3 levels)	TSH associated with TC, LDL-C, DBP and TG, MetS	[30]
Kim, 2009	44,196, 25,147 M	25–70 yrs	Yes	Age	Men: FT4 associated with BP, HDL-C, FBG, TG, and with WC in those over 50; Women: associated with BP, HDL-C, FBG, WC; no association with MetS, when adjusted for age	[4]
Garduno-Garcia, 2010	3033	18–70 yrs	No	Age, sex	TSH associated with TC, TG, WC; FT4 associated with HDL-C, WC, HOMA-IR; 10% had SCH.	[31]
Ruhla, 2010	1333, 481 M	>18 yrs	No	Age	TSH positively associated with BMI, TG, MetS; no data on FT4 or FT3	[32]
Park, 2011	5998, 3469 M	>18 yrs	No	Age, sex, BMI, smoking (y/n), alcohol (y/n), exercise (y/n)	TSH and FT4 associated with WC, FBG, HDL-C, FT4 also with DBP; higher TSH predicted MetS at follow-up	[5]
Tarcin, 2012	211, 24 M	Obesity, 18–73 yrs	No	Age	FT3/FT4 negatively associated with FBG, TG, BP, TT3 positively with HOMA-IR, FBG, WC	[33]
Waring, 2012	2119	70–79 yrs	No	Age, sex, race, BMI, smoking, HOMA-IR	TSH associated with MetS prevalence, in entire cohort with broad range of TSH levels, and in euthyroid range	[34]
Oh, 2013	2760	18–39	Women only	Age, BMI, HOMA-IR	TSH between 2.5 and 4.5 associated with BMI, WC, BP, TG	[35]
Roef, 2014	2315, 1177 M	Middle-aged	No	Age, sex, height, current smoking	FT3 and FT3/FT4 ratio positively associated with BMI, WC, TG, BP, FBG, negatively with HDL-C; FT4 negatively associated with BMI, WC, TG	[36]
Mehran, 2014	3755, 1709 M	≥20 yrs	No	Age, sex, smoking, BMI, HOMA-IR	FT4 associated with HDL-C, LDL-C, TG, WC, BP, but not fasting glucose; no FT3 data	[37]
Minami, 2015	283, 161 M	Children, 6–15 yrs	Yes	None	Higher FT3/FT4 ratio in boys with MetS; no difference thyroid function in girls with MetS	[38]
Laclaustra, 2015	3533 M	20–65 yrs	Men only	Age, alcohol consumption, smoking (never, former, current)	Higher TSH & lowest FT4 quintiles associated with higher MetS prevalence	[39]
Kim, 2016	13,496, 8168 M	Middle-aged, 35–65 yrs	No	Age, sex, % body fat, smoking, HOMA-IR	Higher quartiles of T3 and T3/T4 ratio associated with MetS; only measured total T4 and T3 levels; modified MetS criteria	[6]
Kim, 2017	12,037, 6950 M	Middle-aged, 35–65 yrs	No	Age, sex, smoking	Highest T3 quartile associated with highest 6-year MetS incidence; only measured total T4 and T3 levels; modified MetS criteria	[21]
Ferrannini, 2017	940	30–60 yrs	No	Age, sex, BMI, WHR, family history of diabetes	Higher FT3 associated with decreased insulin sensitivity (insulin clamp), predicted follow-up increases in glycaemia	[22]
Park, 2017	132,346, 66,991 M	>18 yrs	Yes	Age, BMI, smoking status, menopausal status (in women)	FT3FT4 ratio associated with increased risk of MetS parameters and insulin resistance	[7]

*BMI* body mass index, *BP* blood pressure, *DBP* diastolic blood pressure, *FBG* fasting blood glucose, *HDL-C* high-density-lipoprotein-cholesterol, *HOMA-IR* homeostasis-model assessment - insulin resistance, *LDL-C* low-density-lipoprotein-cholesterol, *M* males, *MetS* metabolic syndrome, *SCH* subclinical hypothyroidism, *TC* total cholesterol, *TG* triglycerides, *WC* waist circumference, *WHR* waist-to-hip ratio, *yrs.* years, *y/n* yes/no

There are a number of possible explanations for the relationship between FT3 and the different MetS components, c.q. insulin resistance. One may be that a small increase of T3 may directly influence insulin sensitivity. Recent data have supported this with the notion that overproduction of thyroid hormones may have a negative effect on peripheral insulin action [22]. Indeed, it has been shown that in obese individuals, who more frequently are insulin-resistant, FT4 levels are lower, and TSH and FT3 levels are higher. This has been postulated to be the consequence of an excess of nutrition, especially when consuming a diet high in fat. Obesity may directly influence tissue type 2 deiodinase activity -which converts T4 into T3- in order to compensate for this. It has also been argued that in the brain T3 may have a stimulatory effect on food intake behaviour, which can be considered a compensation for the increase of metabolic rate which is induced by the thyroid hormone itself [23]. This author suggested that T3 may stimulate specific neurons that influence appetite-increasing neuropeptides. However, in our data we did observe lower FT4 and higher TSH levels with increasing BMI, but no significant changes of FT3. However, changes in brain T3 production may not be mirrored by similar changes in plasma FT3. Another explanation may be that MetS or insulin resistance itself may lead to a relative higher intracellular production of T3 as a way to compensate -on a local tissue level- for the disturbances in the metabolic state [24, 25]. The increase in local T3 production may be intended to limit nutrient overload by inducing tissue thermogenesis and simulating metabolic activity [25, 26]. It has also been postulated that 3,5-diiodo-l-thyronine (T2) has marked effects on energy metabolism in order to protect skeletal muscle against excessive intramyocellular lipid storage [27], that may occur in MetS. A positive energy balance both as a consequence of caloric excess and due to insufficient physical activity may contribute to this. In contrast, caloric restriction and prolonged fasting have been associated with lower type 2 deiodinase activity, which will reduce the conversion of T4 to T3 and lower plasma (F)T3 levels, and an increase in the conversion to reverse T3. In addition, the slightly lower (F)T4 levels observed in obesity themselves may upregulate tissue type 2 deiodinase activity, thereby leading to a relative increase of T3. Other factors which may influence tissue deiodinase activity and thereby resulting T3 levels are changes in circadian rhythm. Indeed, it has been shown that disturbances in sleep pattern may play a role in the development of obesity and diabetes [28, 29].

Our study has several strengths, but also some weaknesses. It comprises a large dataset of participants from the general population with available thyroid hormone parameters. Also, all subjects have been uniformly characterized, with well-standardized BP measurements and blood drawing in the fasting state, thereby minimizing the effect of possible circadian changes in thyroid hormone parameters. The large number of participants allowed to assess all relationships for men and women separately, which is of importance considering the significant differences between sexes for a large number of baseline characteristics (Table 1). As mentioned, this is one of the few studies in which confounding factors like smoking and alcohol have been taken into account. Currently, we lack information regarding menopausal status of the female participants, which may influence the possible difference between sexes. Also, data on physical activity could not be taken into account, and it is known that this factor may directly influence some MetS components, and is an important factor in maintaining an optimal energy balance. Tests to measure anti-thyroid peroxidase antibody levels were not available. Due to the cross-sectional design, we can not evaluate the effects of thyroid hormone parameters on future development of MetS. This also relates to the fact that the LifeLines study did not systematically collect medication use during follow-up investigations.

## Conclusions

In conclusion, plasma levels of both FT4 and FT3 are associated with several components of the MetS, after correction for possible important confounding factors. In the highest FT3 and FT3FT4 quartiles there is a 50–80% increased risk of having MetS compared to the lowest quartile. Further studies are needed to assess the possible causality of this relationship.

## Additional files

Additional file 1: Table S1. Thyroid hormone parameters and components of the metabolic syndrome – men. Thyroid hormone parameters and components of the metabolic syndrome for TSH, FT4, TF3 and FT3FT4 quartiles, respectively, separately for men only. (DOCX 11 kb)

Additional file 2: Table S2. Thyroid hormone parameters and components of the metabolic syndrome – women. Thyroid hormone parameters and components of the metabolic syndrome for TSH, FT4, TF3 and FT3FT4 quartiles, respectively, separately for women only. (DOCX 11 kb)

## Abbreviations

BMI: Body mass index
BP: Blood pressure
FBG: Fasting blood glucose
HbA1c: Glycated haemoglobin
HDL-C: High-density lipoprotein cholesterol
LDL-C: Low density lipoprotein cholesterol
MetS: Metabolic syndrome
OR: Odds ratio
TC: Total cholesterol
TG: Triglycerides
WC: Waist circumference
