# Supplementary material for: Thyroid function and metabolic syndrome in the population-based LifeLines cohort study
Source: BMC Endocr Disord. 2017 Oct 16;17:65. doi: 10.1186/s12902-017-0215-1 (PMC5644133; doi:10.1186/s12902-017-0215-1)
Supplement: Supplementary file 1 — Thyroid hormone parameters and components of the metabolic syndrome – men. Thyroid hormone parameters and components of the metabolic syndrome for TSH, FT4, TF3 and FT3FT4 quartiles, respectively, separately for men only. (DOCX 11 kb) [file 12902_2017_215_MOESM1_ESM.docx]

Supplemental Table 1. Thyroid hormone parameters and components of the metabolic syndrome – men

TSH quartiles Q1 Q2 Q3 Q4 P-value

Elevated blood pressure 55.5 56.1 54.9 55.5 NS

Elevated blood glucose 18.4 18.3 18.0 17.8 NS

Low HDL-cholesterol 22.9 21.1 22.0 23.8 NS

Elevated triglycerides 21.8 22.8 24.5 27.3 <0.001

Elevated waist circumference 27.4 26.3 27.0 25.5 NS

% with metabolic syndrome 19.8 20.0 21.0 21.8 NS

FT4 quartiles Q1 Q2 Q3 Q4 P-value

Elevated blood pressure 60.0 56.7 52.3 53.1 <0.001

Elevated blood glucose 22.7 19.1 16.7 14.2 <0.001

Low HDL-cholesterol 26.5 21.3 22.3 19.7 <0.001

Elevated triglycerides 30.2 24.6 21.6 20.2 <0.001

Elevated waist circumference 33.4 27.4 23.6 21.9 <0.001

% with metabolic syndrome 27.7 21.2 17.7 16.3 <0.001

FT3 quartiles Q1 Q2 Q3 Q4 P-value

Elevated blood pressure 56.7 55.3 54.4 55.8 NS

Elevated blood glucose 19.7 18.2 18.0 16.7 NS

Low HDL-cholesterol 19.2 21.1 24.7 24.6 <0.001

Elevated triglycerides 22.1 24.3 24.8 25.0 NS

Elevated waist circumference 26.5 25.0 28.3 26.4 NS

% with metabolic syndrome 19.4 19.8 21.8 21.6 NS

FT3FT4 quartiles Q1 Q2 Q3 Q4 P-value

Elevated blood pressure 53.4 53.6 56.4 58.5 <0.001

Elevated blood glucose 15.2 16.7 20.3 20.3 <0.001

Low HDL-cholesterol 17.7 20.8 24.3 27.0 <0.001

Elevated triglycerides 18.4 22.6 26.9 28.5 <0.001

Elevated waist circumference 23.0 22.4 27.8 33.1 <0.001

% with metabolic syndrome 15.5 17.8 22.5 26.7 <0.001
