# Supplementary material for: Thyroid function and metabolic syndrome in the population-based LifeLines cohort study
Source: BMC Endocr Disord. 2017 Oct 16;17:65. doi: 10.1186/s12902-017-0215-1 (PMC5644133; doi:10.1186/s12902-017-0215-1)
Supplement: Supplementary file 2 — Thyroid hormone parameters and components of the metabolic syndrome – women. Thyroid hormone parameters and components of the metabolic syndrome for TSH, FT4, TF3 and FT3FT4 quartiles, respectively, separately for women only. (DOCX 11 kb) [file 12902_2017_215_MOESM2_ESM.docx]

Supplemental Table 2. Thyroid hormone parameters and components of the metabolic syndrome – women

TSH quartiles Q1 Q2 Q3 Q4 P-value

Elevated blood pressure 30.1 30.2 30.8 33.4 NS

Elevated blood glucose 8.0 8.0 9.8 10.6 <0.001

Low HDL-cholesterol 19.1 18.5 17.3 18.7 NS

Elevated triglycerides 7.0 8.6 8.4 9.7 <0.001

Elevated waist circumference 42.1 42.3 42.5 42.7 NS

% with metabolic syndrome 10.4 11.5 12.1 13.2 NS

FT4 quartiles Q1 Q2 Q3 Q4 P-value

Elevated blood pressure 30.5 30.1 30.9 33.1 NS

Elevated blood glucose 9.6 8.3 9.2 9.5 NS

Low HDL-cholesterol 19.5 17.3 17.9 19.0 NS

Elevated triglycerides 11.2 8.1 7.2 7.0 <0.001

Elevated waist circumference 47.4 42.2 40.8 39.0 <0.001

% with metabolic syndrome 13.8 11.2 11.7 10.5 <0.001

FT3 quartiles Q1 Q2 Q3 Q4 P-value

Elevated blood pressure 29.2 32.3 31.7 30.8 NS

Elevated blood glucose 8.8 8.6 10.0 9.1 NS

Low HDL-cholesterol 13.5 16.8 20.2 22.7 <0.001

Elevated triglycerides 8.8 7.5 9.0 8.5 NS

Elevated waist circumference 42.0 42.7 42.9 41.8 NS

% with metabolic syndrome 10.2 11.1 13.4 12.4 <0.001

FT3FT4 quartiles Q1 Q2 Q3 Q4 P-value

Elevated blood pressure 33.2 30.5 29.7 31.2 NS

Elevated blood glucose 9.9 7.8 8.5 10.3 <0.001

Low HDL-cholesterol 14.2 17.3 19.5 22.5 <0.001

Elevated triglycerides 6.7 7.8 8.0 11.2 <0.001

Elevated waist circumference 40.6 39.8 41.3 47.8 <0.001

% with metabolic syndrome 10.0 10.5 11.8 14.9 <0.001
